# Assessment of Canadian Syncope Risk Score in the prediction of outcomes of patients with syncope at the Emergency Department of Suez Canal University: STROBE compliant

**DOI:** 10.1097/MD.0000000000029287

**Published:** 2022-06-24

**Authors:** Bassant Sayed Moussa, Mohamed Amin Ali, Ahmed Abd El-Nasser Ali, Ahmed EL Sayed Mohammed Abou Zeid

**Affiliations:** a Emergency Medicine, Department Faculty of Medicine, Suez Canal University, Egypt.; b Lecturer of Emergency Medicine Department Faculty of Medicine, Suez Canal University; c Resident of Emergency Medicine Department,Faculty of Medicine, Suez Canal University; d Associate Professor of Emergency Medicine Department, Faculty of Medicine, Suez Canal University.

**Keywords:** MASS, RIPASA

## Abstract

Syncope is a temporary loss of consciousness usually related to insufficient blood flow to the brain. It's also called fainting or "passing out.” Syncope is responsible for 3% to 5% of emergency department visits, with a hospitalization rate in about 40% of cases, with an average stay of 5.5 days. The Canadian Syncope Risk Score showed good discrimination and calibration for 30-day risk of serious adverse events after disposition from the emergency department.

The aim was to assess Canadian Syncope Risk Score in predicting outcomes and mortality at the emergency department of Suez Canal University Hospitals.

A prospective observational cohort study was carried out in emergency department in Suez Canal University Hospital. After approval by the Ethical and Research Committee of Faculty of Medicine, Suez Canal University, 60 patients with syncope attending to emergency department were included to this study. All included participants were assessed by history taking and they also assessed by the Canadian Syncope Risk Score.

The Canadian Syncope Risk Score's mean of the study group was 4.9 and the range of the scores was from −2 to 11. The mean of the percentage of risk of serious events at 30 days in the study group was 29.17% and it ranged from 0.7% to 83.6%.There was a statistically significant difference between means Canadian Syncope Risk Score's score regarding complication occurrence. Cases which showed complications had a mean score of 7.33 compared to a mean score of 1.25 in case of no complication occurrence *P*-value <.001. At a cut-off point of more than 3 for the Canadian Syncope Risk Score's, sensitivity of that score in complication's occurrence prediction was 100% and the specificity was 87.5% *P*-value <.001.

The Canadian Syncope Risk Score's is strong predictor for risk of serious adverse events and a good indicator for admission, with 100% sensitivity and 87.5% specificity at cut off point more than 3.

## 1 Introduction

Syncope is a temporary loss of consciousness usually related to insufficient blood flow to the brain. It's also called fainting or “passing out.” Syncope is a common medical problem, with a frequency between 15% and 39%. In the general population, the annual number episodes are 18.1 to 39.7 per 1000 patients, with similar incidence between genders. This framework is secondary to cerebral hypoperfusion, with short duration (average 12 seconds).^[1]^

In the general population, the annual number episodes are 18.1 to 39.7 per 1000 patients, with similar incidence between genders, and with high prevalence between 10 and 30 years of age, mainly of vasovagal syncope.^[2]^ The first report of the incidence of syncope is 6.2 per 1000 person-years. However, there is a significant increase in the incidence of syncope after 70 years of age, with 5.7 episodes/1000 individuals per year between 60 and 69 years old and with 11.1 episodes/1000 individuals per year between 70- and 79-years age. After 80 years, the annual incidence may reach 19.5 per 1000 individuals.^[3]^

The most commonly considered 7-day outcomes are mortality, dysrhythmias, myocardial infarction, stroke, and rehospitalization. The most commonly considered 30-day outcomes are mortality, hemorrhage requiring blood transfusion, dysrhythmias, myocardial infarction, pacemaker or implantable defibrillator implantation, stroke, pulmonary embolism, and syncope relapse.^[4]^

Thiruganasambandamoorthy et al, prospectively enrolled adults (age ≥ 16 years) with syncope who presented within 24 hours after the event to 1 of 6 large emergency departments from September 29, 2010 to February 27, 2014. then collected standardized variables at index presentation from clinical evaluation and investigations. Adjudicated serious adverse events included death, myocardial infarction, arrhythmia, structural heart disease, pulmonary embolism, serious hemorrhage and procedural interventions within 30 days.^[5]^

Thiruganasambandamoorthy et al, reported that the Canadian Syncope Risk Score showed good discrimination and calibration for 30-day risk of serious adverse events after disposition from the emergency department. Once validated, the tool will be able to accurately stratify the risk of serious adverse events among patients presenting with syncope, including those at low risk who can be discharged home quickly.^[6]^

The Canadian Syncope Risk Score (CSRS) is the latest syncope decision rule to be developed; nine predictors were derived, encompassing clinical evaluation, investigations and likely ED diagnosis to produce a patient risk score between −3 and 11. Risk scores are grouped into risk categories based on the likelihood of serious adverse events (SAEs).^[6]^

### 1.1 Aim of the study

Our study aimed to determine the ability of the CSRS to predict 30-day serious outcomes in patients presenting to the emergency department of Suez Canal university teaching hospitals (ED) with syncope.

## 2 Patient and methods

A prospective observational cohort study was carried out in emergency department in Suez Canal University Hospital. After approval by the Ethical and Research Committee of Faculty of Medicine, Suez Canal University(reference: research #4290) (chairman of the committee:professor dr. Amani Waheed), 60 patients with syncope attending to emergency department were included to this study.

The sample size was calculated using the following formula^[7]^:


$$n={\left[\frac{Z_{\alpha /2}}{\text{E}}\right]}^{2}*\frac{S_{n}\left(1-S_{n}\right)}{\left(\text{P}\right)}$$

where n = sample size, Zα/2 = 1.96 (The critical value that divides the central 95% of the Z distribution from the 5% in the tail), P = Prevalence/proportion of disease = 16.8%,^[5]^ Sn = Sensitivity = 97.8%,^[5]^ and E = Margin of error/width of confidence interval = 5.5%.

So, by calculation, the sample size is equal to 60 subjects after addition of 10% drop-out proportion.

All included participants were assessed by history taking and they also assessed by the Canadian Syncope Risk Score. Patients were followed up by phone at 30 days after their index presentation to determine the occurrence of SAE. A 30-day SAE was recorded if any of the following occurred during follow up: death, arrhythmia, myocardial infarction, serious structural heart disease, aortic dissection, pulmonary embolism, severe pulmonary artery hypertension, subarachnoid hemorrhage, significant hemorrhage, any other serious condition or procedural intervention used to treat syncope. Patients unable to be contacted were considered lost to follow up and local heath databases and death registry checked. Patients were followed up by phone and when they returned back to outpatient clinic to assess their conditions.

### 2.1 One month duration follow up

We followed up the patients over one month duration from entering the emergency department with syncope. First we contacted the patients by phone and they were assessed by coming to regular visits to the outpatient clinic during the 30 days.

### 2.2 Statistical analysis

Statistical analyses were performed using Statistical Package for the Social Sciences for Windows version 22. The numerical variables was expressed in terms of mean and standard deviation (Mean ± SD), and qualitative variables were expressed in terms of frequency and frequency percentage. The normality of variables was tested with the Kolmogorov–Smirnov test. Chi-squared test was used for categorical data. Results were expressed as mean ± SD or n, % and *P* < .05 was considered significant.

## 3 Results

The present study showed that age's mean is (55.9 ± 8.30) years old, males represented 51.7% as shown in Table 1. Complications occurred the most in the 50 to 60 years old age group as shown in Table 2.

**Table 1:** Demographic data of the study group (n = 60).

Demographic data		Study group (n = 60)
Age (y)	Mean ± SD	55.9 ± 8.30
	Range	40–72
Male	N (%)	28 (46.7)
Female	N (%)	32 (53.3)
Statistics	Test	Chi-squared
	*P*-value	.606
Male	Mean ± SD	55.57 ± 9.07
Female	Mean ± SD	56.19 ± 7.69
Statistics	Test	Mann–Whitney *U*
	*P*-value	.561

*P*-value is significant if <.05. Table 1 shows that ages of patients in the study ranged from 40 to 72 years with a mean age of 55.9 years old. Males represented 51.7% with a mean age of 55.57 years old and females represented 48.3% of the study with a mean age of 56.19 years old.

**Table 2:** Age classification of the study group (n = 60).

			Score				Complications	
Age groups	N	%	Mean	Maximum	Median	Minimum	N	%
40–50 y	24	40	2.75	11.0	1.5	−2.0	9	37.5%
50–60 y	18	30	6.00	11.0	5.0	3.0	15	83.3%
60–70 y	12	20	6.50	11.0	6.0	3.0	9	75.0%
70–80 y	6	10	7.00	9.0	7.0	5.0	3	50.0%
Total	60	100	2.75	11.0	1.5	−2.0	36	60%
Chi-squared *P*-value			Independent samples median test		***<.001***	Chi-squared *P*-value	.426	
*P*-value is significant if <.05.								

Within the study population, the majority of patients were within age range of 40 to 50 years old. The maximum score recorded was 11 and the minimum score recorded was negative two. Complications occurred the most in the 50 to 60 years old age group. The frequency of the aforementioned was 83.3%. There is significant difference between age groups and CSRS score.

The CSRS's mean of the study group was 4.9 and the range of the scores was from −2 to 11. And very high-risk cases had the highest distribution (35%), while cases with very low risk had lowest distribution (10%).

The most frequent complication which occurred within 30 days in this study was uncontrolled atrial fibrillation, followed by myocardial infarction and Supraventricular tachycardia and the least frequent were gastrointestinal bleeding and pulmonary embolism; there was a statistically significant difference between means *Risk of serious adverse event score* regarding complication occurrence. With higher scores in Cases which showed *complication.*

Also, we found 46.7% of cases was admitted, and those who were admitted, had mean CSRS score significantly higher than cases of on-site patient discharge, and Cases which showed complications had a significantly higher CSRS score than in case of no complication occurrence.

At cut-off point of more than 3 for the CSRS, sensitivity of that score in complication's occurrence prediction was 100% and the specificity was 87.5% meaning that the score is better in ruling out the complications’ occurrence. The receiver operating characteristic (ROC) curve showed a significant area under curve (AUC) of 0.974 as shown in Table 3.

**Table 3:** Predictive value of CSRS score for very high risk of serious adverse events by receiver operating characteristic curve analysis.

CSRS	Very high risk
Positive group (N, %)	21 (35)
Negative group (N, %)	39 (65)
Cut off level	>5
Area under curve	1
Sensitivity (%)	100
Specificity (%)	100
*P*-value	<.0001^∗^
95% confidence interval	0.940–1.000
Prevalence (%)	35
Positive predictive value	100
Negative predictive value	100

At a cut off level of CSRS > 5, there is statistically significant predictive value for very high risk serious adverse events (*P* < .05), with sensitivity 100% and Specificity 100%. CSRS = Canadian Syncope Risk Score.

∗ *P* < .05 is considered significant.

## 4 Discussion

The CSRS was developed to predict 30-day serious outcomes; it consists of three parameters; (1) Clinical evaluation ”Predisposition to vasovagal symptoms, history of heart disease and Any systolic pressure reading <90 or >180 mm Hg” (2); Investigations; “Elevated troponin level (>99th percentile of normal population), abnormal QRS axis (<–30° or >100°), QRS duration >130 ms, and Corrected QT interval >480 ms” and (3); Diagnosis in emergency department; Vasovagal syncope or Cardiac syncope, its score ranged between (−3 to 11) for interpretation A score of −2 or lower confers a very low risk (<1%), scores of −1 to 3 confer a low to medium risk (1%–8%), and scores of 4 or more confer a high or very high risk (>12%).^[6]^

Our study aimed to determine the ability of the CSRS to predict 30-day serious outcomes in patients presenting to the emergency department of Suez Canal university teaching hospitals (ED) with syncope.

### 4.1 Demographic data

The current study enrolled 60 patients with acute syncope, it shows that the mean of age is (55.9 ± 8.30) years old as shown in Table 1. Males represented 51.7%, Complications occurred the most in the 50 to 60 years old age group as shown in Table 2. This result in line with Chan et al, 2020 prospective observational study in Single center in Brisbane, Australia that enrolled 283 patients found that the average age was 55.6 years (SD 22.7) with 37.1% being male^[8]^ and Thiruganasambandamoorthy et al, 2016 study that enrolled 4030 patients at 6 large emergency departments in teaching hospitals in 4 Canadian cities, regarding age but differ with them as they found female represent higher prevalence.^[6]^

### 4.2 Score results

In the current study the CSRS's mean of the study group was 4.9 and the range of the scores was from −2 to 11.

Our study showed that very high-risk cases had the highest distribution (35%), while cases with very low risk had lowest distribution (10%). Our results in line with Zimmermann et al, 2020 who found The rate of observed serious outcomes within 30 days increased from 0.8% in the very low risk group (CSRS equal to or below −2) to 48% in the (very) high risk group (CSRS equal to or above 4, Hazard ratio 79.4, 95% CI 11.1–570.9).^[9]^

In other hand, there were 141 (49.8%) very-low-risk, 62 (21.9%) low-risk, 61 (21.6%) medium-risk, 12 (4.2%) high-risk and 7 (2.5%) very-high-risk patients identified in Chan et al, 2020 study.^[8]^

### 4.3 Relation between scores and s serious adverse event

We defined a serious adverse event as the detection or occurrence of any serious condition related to syncope within 30 days after disposition from the emergency department. The composite outcome included any of the following serious adverse events: death, arrhythmia, myocardial infarction, serious structural heart disease, aortic dissection, pulmonary embolism, severe pulmonary hypertension, severe hemorrhage, subarachnoid hemorrhage, any other serious condition causing syncope and procedural interventions for the treatment of syncope.

The present study found the most frequent complication which occurred within 30 days in this study was uncontrolled atrial fibrillation (22.2%), followed by myocardial infarction (16.7%), supraventricular tachycardia (16.7%) and the least frequent were gastrointestinal bleeding (8.3%) and pulmonary embolism (8.3%).

Ragan and Lin, 2021 study showed that; in the low-risk groups, 0.3% of very low risk and 0.7% of low-risk patients experienced any serious 30-day outcome with no ventricular arrhythmias or deaths observed. In the highest risk group, 51.3% of patients experienced any serious outcome with 7 deaths and 33 arrhythmias observed.^[10]^

### 4.4 Admission and serious adverse events

In the present study; 46.7% of cases was admitted, and those who were admitted, had mean CSRS score significantly higher than discharged cases, and cases which showed complications had a significantly higher CSRS score than in cases with no complication

Thiruganasambandamoorthy et al, 2020 study found that the short-term serious morbidity and mortality for ED syncope was very low, with 0.3% risk for each of 30-day mortality and ventricular arrhythmia, as previously reported.^[5,11]^

Additionally, none of the patients in these categories died or experienced ventricular arrhythmia. Hence, they believe that these patients can be discharged quickly after ED evaluation.^[5]^ Our results inconsistence with Thiruganasambandamoorthy et al, 2020 who found a statistically significant difference in between the distributions of complication occurrence regarding on-site admission of patients. Admitted cases with complications’ occurrence were 31 compared to only one case which had complications although not admitted.^[5]^

The difference between these results illustrated as our population is hospital based and it is only a small fraction of patients from the general population that presents in a clinical setting.

### 4.5 Validity of CSRS

For validation in the current study; we found at cut-off point of more than 3 for the CSRS, sensitivity of that score in complication's occurrence prediction was 100% and the specificity was 87.5% meaning that the score is better in ruling out the complications’ occurrence. The ROC curve showed a significant AUC of 0.974 as shown in Table 3.

The correlations between risk of serious adverse events and both CSRS and risk category were strong positive statistically significant. We agree with (Thiruganasambandamoorthy et al, 2020). In a recent metacentric study, enrolled 3819 patients they found (0.3%) patients at very low risk and (0.7%) patients at low risk experienced 30-day serious outcomes, and this proportion significantly increased to 40 of 78 (51.3%) total patients in the very-high-risk group. There were similar steady significant increases in the subtypes of serious outcomes from the very-low-risk to the very-high-risk categories.^[5]^

All these finding illustrated that CSRS is strong predictor for risk of serious adverse events and a good indicator for admission.

Many studies discussed the validity of CSRS as Thiruganasambandamoorthy et al, 2020 study who reported an AUC of 0.88^[5]^ (the rest of validation results in the study).

Ragan and Lin, At a threshold score of −1, the CSRS performed with a sensitivity of 97.8% (95% CI 93.8%–99.6%) and a specificity of 44.3% (95% CI 42.7%–45.9%). The AUC of the model was 0.91 (95% CI 0.88–0.93).^[10]^

In other study conducted by Thiruganasambandamoorthy et al, 2018 The accuracy of the CSRS remained high with area under the ROC curve at 0.87 (95% CI 0.82–0.92), similar to the derivation phase (0.87; 95% CI 0.84–0.89). The score showed excellent calibration at the prespecified risk strata. For the very-low risk category (0.3% SAE of which 0.2% were arrhythmia and no deaths) the sensitivity was 97.5% and negative predictive value was 99.7% (95% CI 98.7–99.9). For the very high-risk category (61.5% SAE of which 26.9% were arrhythmia and 11.5% death) the specificity was 99.4% and positive predictive value was 61.5% (95% CI 43.0–77.2).^[12]^

Chan et al, 2020 mentioned that for a threshold of −1 or higher there were 137 false positive cases and for a threshold of 1 or higher there were 75. The number of false negative cases (n = 2) did not change with the different thresholds examined. The CSRS had a sensitivity of 71.4% and specificity of 50.4% for a threshold score of–1 or higher. The CSRS performed with the same sensitivity for a threshold score of 1 or higher but with higher specificity of 72.8%.^[8]^

### 4.6 Limitations of the study

The low prevalence of 30-day SAEs in our study and syncope patients in general has been acknowledged previously as one of the challenges in validating any syncope clinical decision rule.

## 5 Conclusion

- Syncope accounting for 1% to 3% of all emergency department visits.
- The ages mean is (55.9 ± 8.30) years old. Males represented 51.7%.
- Most prevalent type of syncope is cardiac syncope represent about 65%.
- The CSRS is strong predictor for risk of serious adverse events and a good indicator for admission, with 100% sensitivity and 87.5% specificity at cut off point more than 3, for complication's prediction, meaning that the score is better in ruling out the complications occurrence.
- There was a statistically significant difference between means CSRS score regarding complication occurrence. Cases which showed complications had a mean score of 7.33 compared to a mean score of 1.25 in case of no complication occurrence.

## 6 Recommendations

- Based on the study results, we recommend that patients with very low- risk and low-risk CSRS can be discharged.
- Patients at medium risk be involved in a shared decision approach regarding disposition.
- Patients at high risk should be hospitalized for a short course.
- Implementation of the CSRS will improve patient safety and reduce health care resource use unnecessary investigations and unneeded hospitalization.

## Author contributions

**Supervision:** Ahmed Abou zeid, Bassant Mousa, Bassant Moussa, Mohamed Ali.

**Writing – original draft:** Ahmed Abdelnasr, Ahmed Ali.

**Writing – review & editing:** Bassant Mousa, Bassant Moussa.
